# The role of autophagy in the cross-talk between epithelial-mesenchymal transitioned tumor cells and cancer stem-like cells

**DOI:** 10.1186/s12943-016-0573-8

**Published:** 2017-01-30

**Authors:** Fabrizio Marcucci, Pietro Ghezzi, Cristiano Rumio

**Affiliations:** 1 Dipartimento di Scienze Farmacologiche e Biomolecolari, University of Milano, via Trentacoste 2, 20133 Milan, Italy; 2 Brighton & Sussex Medical School, Trafford Centre, University of Sussex, Falmer, Brighton, BN1 9RY UK

**Keywords:** Metastasis, Resistance, Cycling, Autophagic, Tumor propagation, Tumor microenvironment

## Abstract

Epithelial-mesenchymal transition (EMT) and cancer stem-like cells (CSC) are becoming highly relevant targets in anticancer drug discovery. A large body of evidence suggests that epithelial-mesenchymal transitioned tumor cells (EMT tumor cells) and CSCs have similar functions. There is also an overlap regarding the stimuli that can induce the generation of EMT tumor cells and CSCs. Moreover, direct evidence has been brought that EMT can give rise to CSCs. It is unclear however, whether EMT tumor cells should be considered CSCs or if they have to undergo further changes. In this article we summarize available evidence suggesting that, indeed, additional programs must be engaged and we propose that macroautophagy (hereafter, autophagy) represents a key trait distinguishing CSCs from EMT tumor cells. Thus, CSCs have often been reported to be in an autophagic state and blockade of autophagy inhibits CSCs. On the other hand, there is ample evidence showing that EMT and autophagy are distinct events. CSCs, however, represent, by themselves, a heterogeneous population. Thus, CSCs have been distinguished in predominantly non-cycling and cycling CSCs, the latter representing CSCs that self-renew and replenish the pool of differentiated tumor cells. We now suggest that the non-cycling CSC subpopulation is in an autophagic state. We propose also two models to explain the relationship between EMT tumor cells and these two major CSC subpopulations: a branching model in which EMT tumor cells can give rise to cycling or non-cycling CSCs, respectively, and a hierarchical model in which EMT tumor cells are first induced to become autophagic CSCs and, subsequently, cycling CSCs. Finally, we address the therapeutic consequences of these insights.

## Background: Epithelial-mesenchymal transition and cancer stem-like cells

Epithelial-mesenchymal transition (EMT), i.e., the conversion of cells with an epithelial phenotype into cells with a mesenchymal phenotype [1, 2] involves changes that lead to loss of cell–cell adhesion and cell polarity. EMT is critical for embryonic development. In adults it occurs during wound healing, tissue regeneration, organ fibrosis, and tumor progression. Epithelial-mesenchymal transitioned tumor cells (EMT tumor cells) have been reported to possess increased motility and invasiveness, tumor-propagating potential, and resistance to apoptosis and antitumor drugs [3, 4].

CSCs are a subpopulation of tumor cells that have high tumor-propagating potential [5], enhanced metastasis-forming potential [6] and are resistant to antitumor drugs [7]. There is a large overlap in the characteristics of EMT tumor cells and CSCs also as regards the stimuli that can induce the generation of EMT tumor cells and CSCs. Thus, both are the result of two main events. The first is the genetic and epigenetic instability of tumor cells [8–12]. The second event is represented by stimuli from the tumor microenvironment (TME) that promote a cross-talk between different cell types within the TME and that is largely effected by paracrine factors that are released in response to the stimuli and interact with their corresponding receptors on tumor cells [13–15]. Ligand-receptor pairs like hepatocyte growth factor/c-MET, transforming growth factor (TGF)-β/TGF-β receptor, interleukin-6 (IL-6)/IL-6 receptor, platelet-derived growth factor (PDGF)/PDGF receptor (PDGFR), epidermal growth factor (EGF)/EGF receptor, fibroblast growth factor (FGF)/FGF receptor (FGFR), Gas6/AXL, WNT/Frizzled, Hedgehog/Smoothened and Notch ligands/Notch are examples of paracrine factors and receptors that have been shown to promote the induction of both EMT and CSCs [7, 15–20]. Eventually, direct evidence has been brought showing that EMT can give rise to CSCs. Thus, induction of EMT in immortalized human mammary epithelial cells through the ectopic expression of EMT-promoting transcription factors resulted in the acquisition of mesenchymal traits and expression of stem-cell markers [21]. These cells had an increased ability to form mammospheres, a property associated with epithelial stem cells [21]. Further demonstrating the close linkage between EMT and CSCs, it was shown that down-regulation of the receptor tyrosine kinase AXL reversed EMT in human epithelial cells and breast CSCs attenuating self-renewal and restoring chemosensitivity of breast CSCs [16].

Given the multiplicity of genetic and environmental stimuli that are at the origin of EMT and CSCs, it is not surprising that a large number of overlapping intracellular signaling pathways have been reported to be involved in the induction of both. Intracellular signaling hubs like focal adhesion kinase and SRC, pathways like phosphoinositide 3-kinase–AKT–mechanistic target of rapamycin, RAF-RAS-mitogen-activated protein kinase, transcription factors like small mother against decapentaplegic (SMAD), nuclear factor kappa-light-chain-enhancer of activated B cells (NF-κB), signal transducer and activator of transcription (STAT) 3, and reactive oxygen species have been shown being involved in the induction of EMT and CSCs [7, 15, 22–26].

Given the great similarity between the functions, inducing stimuli and intracellular signaling pathways of EMT tumor cells and CSCs, and the observation that EMT can give rise to CSCs, one is led to ask whether EMT tumor cells are identical to CSCs. Answering this question has considerable translational relevance, because EMT and CSCs have become important targets in drug discovery and several anti-EMT and anti-CSC compounds are now in active clinical development [15, 27].

## Main text

### Differences between EMT tumor cells and CSCs

While EMT tumor cells and CSCs are induced by similar stimuli and apparently discharge similar tasks, there is now considerable evidence suggesting that the two cell types are distinct and may represent different stages of a tumor cell dedifferentiation process. Thus, salinomycin, a natural, fused polypyran ionophore, is a widely used anti-coccidiosis agent. It was found to have anti-CSC activity in a chemical screen designed to discover compounds toxic for breast CSCs [28]. In head and neck squamous cell carcinoma stem cells, salinomycin significantly inhibited sphere forming-capability, repressed the expression of CSC markers and reduced invasiveness of CSCs [29]. On the other hand, salinomycin induced the expression of EMT markers and decreased expression of E-cadherin, a hallmark trait of epithelial tumor cells. Thus, salinomycin appears to inhibit CSCs while, at the same time, promoting EMT. Unfortunately, several different mechanisms of action have been ascribed to salinomycin and, therefore, no conclusion can be drawn as to the signaling pathway(s) or factor(s) dictating this switch.

Other work has shown that the homeobox transcription factor paired related homeobox 1 (Prrx1) can be such a switch factor. Prrx1 induced EMT in cooperation with the other transcription factor Twist1, conferring migratory and invasive properties [30]. Loss of Prrx1, on the other hand, was required for cancer cells to metastasize and acquire CSC properties and markers. Importantly, this article showed that, contrarily to common knowledge, functions of EMT tumor cells and CSCs are not coincident. Rather, EMT tumor cells were shown to have migratory and invasive properties, while CSCs metastasized.

It has also been reported that tumor cell subpopulations expressing a strong epithelial gene program are enriched in highly metastatic CSCs, while subpopulations with stable mesenchymal traits (i.e. EMT tumor cells) are impoverished in CSCs, confirming that EMT tumor cells and CSCs are functionally and phenotypically separate entities [31]. The same study showed that both tumor cell subpopulations cooperate so that nonmetastatic cells promote the escape of metastatic cells for metastatic colonization. These results, as well as the previous ones, while suggesting that EMT tumor cells and CSCs are indeed separate entities, do not exclude the possibility that they are developmentally related.

A further turn of complexity to this picture was introduced by the observation that CSCs themselves can undergo an EMT [32]. In fact, in cell lines derived from oral and skin carcinomas, EMT occurred within the CD44^high^ CSC fraction resulting in two CSC phenotypes, one predominantly epithelial with high expression of epithelial specific antigen (ESA), and another with EMT tumor cell characteristics and low expression of ESA. CSCs could switch between these two phenotypes with EMT tumor cells being relatively quiescent [33].

A dichotomy between EMT tumor cells and CSCs has also been confirmed in samples of different subtypes of breast cancers from tumor patients [34]. A method for scoring transcriptomic EMT signatures in different types of cancer showed that tumors of predominantly mesenchymal phenotype do not always show resistance to chemotherapy and suggested that it is the CSC phenotype, rather than the EMT phenotype that engenders drug resistance [35].

Overall, there is now considerable evidence that EMT tumor cells and CSCs are neither phenotypically nor functionally identical. Some of the reports that have been discussed suggest even that EMT tumor cells and CSCs are two (de)differentiation pathways that can cross each other, but are, nevertheless, distinct. This is in apparent contrast with the commonly held view that EMT can lead to the generation of CSCs and that EMT tumor cells and CSCs exert largely overlapping tasks.

### Autophagy as a key trait that distinguishes CSCs from EMT tumor cells

In addition to the differences that have been discussed above, we propose that also macroautophagy (herein, autophagy) [36] is a key trait that distinguishes CSCs from EMT tumor cells. Autophagy is an adaptive catabolic process of cells that stop dividing and enter quiescence, and occurs in response to different forms of environmental stress, including nutrient deprivation, growth factor depletion, and hypoxia [36]. Autophagy involves the delivery of cytoplasmic cargoes sequestered inside double-membrane vesicles to lysosomes. Autophagosomes are then formed and this is where the captured material is degraded. This process of self-digestion provides nutrients to maintain vital cellular functions during fasting and other forms of stress. Autophagy has a suppressor role in initial steps of tumorigenesis, but has a prosurvival effect in established tumors by allowing tumor cells to cope with environmental and therapy-induced stress [36].

CSCs have often been reported to be in an autophagic state [37–39] and blockade of autophagy reduces their activity [40] and sensitizes them to antitumor drugs [41]. Moreover, one of the most commonly used markers for CSCs of several tumor types, CD133, promotes the autophagocytic activity of hepatoma CSCs [42], suggesting a functional link between CSCs and autophagy.

On the other hand, there is ample evidence that EMT and autophagy are distinct and even mutually exclusive events. Thus, autophagy induction impaired migration and invasion by inhibiting EMT in glioblastoma cells through down-regulation of the EMT-promoting transcription factors Snail and Slug [43]. Vice versa, silencing autophagy-inducing proteins restored the mesenchymal phenotype [43]. The Aurora kinase A inhibitor alisertib induced cell cycle arrest and autophagy and suppressed EMT in human pancreatic cancer cells [44]. Vice versa, Aurora kinase A suppressed autophagy and autophagic cell death by activating mechanistic in mTOR signaling in breast cancer cells [45]. Induction of EMT and metastasis upon inhibition of autophagy was observed also in gastric cancer cells [46].

In several instances a molecular cross-talk between the two pathways has been demonstrated to dictate the choice between EMT and autophagy. Thus, suppression of autophagy was shown to promote tumor growth and metastasis through stabilization of the EMT-promoting transcription factor Twist 1 by the selective autophagy substrate p62 that accumulated due to autophagy inhibition [47]. Similarly, autophagy inhibition by EMT in a p62-dependent manner has been reported in normal hepatocytes [48]. In metastatic breast cancer cells, the death-effector domain-containing DNA-binding protein (DEDD) interacted with class III phosphatidylinositol 3-kinase (PI3KC3) to activate autophagy and attenuated EMT [49]. DEDD physically interacted with PI3KC3 and this led to its stabilization and activation, and autophagic degradation of the EMT-promoting transcription factors Snail and Twist.

Altogether, these results suggest that EMT tumor cells and autophagic CSCs are distinct states of dedifferentiation that ensue in response to similar stimuli. Autophagic tumor cells, however, can induce other tumor cells to undergo EMT upon release of EMT-inducing paracrine factors [50, 51]. We suggest this to be a positive feed-back mechanism whereby autophagic CSCs release danger signals (i.e. paracrine factors) that induce an increasing number of tumor cells to enter a state, EMT, allowing them to cope with stress conditions in the TME. This view is consistent with the knowledge that EMT is the result of a cross-talk between tumor cells and tumor-associated cells and is mediated by the release of paracrine factors [14].

While CSCs can be in an autophagic state that appears to be mutually exclusive with that of EMT tumor cells, one main function of CSCs is to self-renew and to differentiate into mature tumor cells. This process implies active proliferation of CSCs. When cells undergo autophagy, however, they stop dividing and become quiescent, a condition opposite to the previous one.

### Proliferating and autophagic CSCs

There is now increasing evidence that CSCs are themselves heterogeneous [52–54]. Phenotypic [55] and functional [56] heterogeneity of CSCs has been described. These characteristics can change over time [57] and differences between CSC subpopulations are not strictly qualitative but, rather, quantitative [58]. These observations suggest that different CSC subpopulations can switch from one to another in a dynamic manner [58].

Of particular relevance in the present context is the observation that CSCs can be distinguished in predominantly non-cycling CSCs and cycling CSCs [54, 58–60]. This is reminiscent of the proliferating and dormant subpopulations of somatic stem cells, with the dormant stem cell pool representing the most primitive stem cells [55]. Cycling CSCs are associated with cytokine production and cytokine receptor expression and this may be causally related with their replicative potential [61].

We propose that autophagic CSCs correspond to the non-cycling CSC subpopulation. To this regard, in squamous cell carcinoma, two CSC subpopulations have been identified on the basis of their capacity to promote tumor growth or invasion and metastasis, respectively. FGFR 1 inhibition reduced tumor growth without blocking metastasis, whereas PDGFR α inhibition reduced invasion and metastasis, but not tumor growth [62]. PDGFR signaling had been previously shown to induce formation of CSCs from non-CSCs [63]. Interestingly, PDGFR signaling has been reported to be an essential promoter of hypoxia-induced autophagy in tumor cells by prolonging the half-life of hypoxia-inducible factor-1α [64]. This lends support to our view that non-cycling CSCs are in an autophagic state.

In squamous cell carcinoma CSCs the choice between cycling and non-cycling state is induced by TGF-β, which bestows the properties of the non-cycling subpopulation [65]. While non-responding CSCs proliferated faster and accelerated tumor growth, TGF-β-responding progenies invaded and showed increased protection against anti-cancer drugs.

Regarding the functions of cycling and non-cycling CSCs, we have already referred to reports suggesting that the cycling subpopulation accelerates tumor growth, while the non-cycling, possibly autophagic one, promotes invasion, migration and metastasis [60, 62, 65]. Several other reports are in accordance with these results [7, 33, 66], some suggesting that the cycling subpopulation has a predominantly epithelial phenotype in contrast to the predominantly mesenchymal phenotype of the non-cycling subpopulation [33, 60]. Moreover, mesenchymal-like breast CSCs were characterized as CD24^−^CD44^+^, primarily quiescent and located at the tumor-invasive front, whereas epithelial-like CSCs expressed aldehyde dehydrogenase, proliferated and were located more centrally within tumors [60].

Regarding the relationship between cycling and non-cycling CSCs, we propose that CSCs develop into one or the other phenotype depending on stimuli from the TME. This view is supported by the observation that CSCs with predominantly epithelial phenotype relied mainly on oxygen metabolism, whereas predominantly mesenchymal CSCs showed decreased mitochondrial mass and membrane potential, consumed less oxygen per cell and produced markedly reduced levels of reactive oxygen species, suggesting that this subpopulation relied mainly on glycolysis for energy production [32]. Importantly, the shift towards a mesenchymal phenotype was induced by stimuli from the TME like hypoxia or tumor necrosis factor. These results are of interest because CSC heterogeneity may well explain the contradictory results on CSC metabolism, with several reports showing oxidative phosphorylation as the predominant energy source, while several others show that CSCs rely on glycolysis for energy production (see, for example, Refs. [67, 68]).

### From EMT tumor cells to CSCs: What roads are taken?

As we have already discussed, it has been shown that EMT can give rise to CSCs [6, 21]. EMT tumor cells and CSCs have also been reported to have similar functions, although some results discussed above contrast this view [30, 31]. Moreover, most recent evidence has put into question that EMT tumor cells can metastasize [69, 70]. Yet, if we accept the view that there are two major subpopulations of CSCs, a cycling and a non-cycling one, then we have to address the issue as to which is the relationship between EMT tumor cells and these CSC subpopulations, and between the CSC subpopulations themselves. We have proposed that cycling and non-cycling CSCs are the result of different classes of cues from the TME. In support, it has been demonstrated that CSCs may display a background of genetic instability that is similar to that of differentiated tumor cells suggesting that, at least in some cases, environmental cues may play the predominant, if not the sole role in giving rise to CSCs [71]. This does not exclude, however, that in other instances stimuli from the TME may interact with a background of genetic instability. On the other hand, we have not addressed the issue as to whether the two subpopulations represent two consecutive steps of a single developmental pathway or if they are the result of two different developmental pathways. Moreover, before illustrating possible models that can accommodate acquired knowledge, it is important to mention that both EMT tumor cells as well as CSCs can derive from and can revert back to differentiated tumor cells [72, 73].

**Fig. 1:**
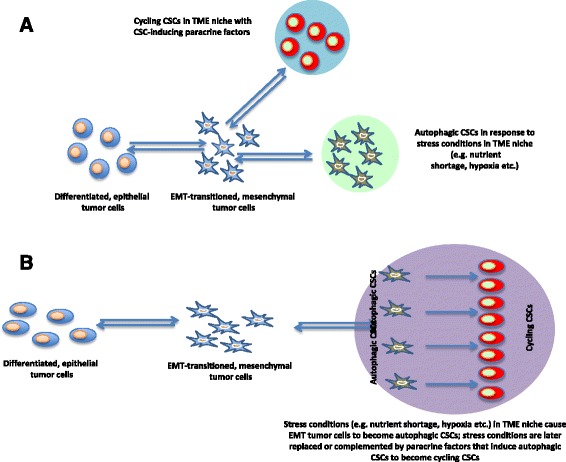
Two Models for EMT Tumor Cells Giving Rise to CSCs. **a**. Branching Model. In this model EMT tumor cells give rise to the cycling CSC compartment in response to paracrine factors within a niche in the TME that is conducive to the development of this CSC subpopulation. Alternatively, EMT tumor cells give rise to the non-cycling, autophagic CSC compartment in response to stress conditions such as nutrient shortage, mechanical stress, hypoxia etc. It is possible that this is a default pathway occurring in the absence of paracrine factors. **b**. Hierarchical Model. Here, EMT tumor cells are induced to become autophagic CSCs in response to stress conditions in the TME. Once these stimuli are relieved and replaced or complemented by paracrine factors, autophagic CSCs become cycling CSCs that self-renew and replenish the pool of differentiated tumor cells. CSC, cancer stem-like cell; EMT, epithelial-mesenchymal transition; TME, tumor microenvironment

Given that knowledge, we propose two models that can explain the relationship between EMT and the two main CSC subpopulations, non-cycling, autophagic CSCs and cycling CSCs. We refer to the first model as the branching model (Fig. 1a). In this model, EMT tumor cells give rise to the cycling CSC compartment in response to paracrine factors within a niche of the TME that is conducive to the development of this CSC subpopulation. Alternatively, EMT tumor cells give rise to the non-cycling, autophagic CSC compartment in response to environmental cues that are represented by stress conditions such as nutrient shortage, mechanical stress, hypoxia etc. It is possible that this is a default pathway that occurs in the absence of paracrine factors. EMT tumor cells that are not diverted into one of the two pathways may remain in their state or may even revert back to differentiated tumor cells.

The second, hierarchical, model (Fig. 1b) is more traditional and has already been proposed for both somatic stem cells as well as CSCs [55, 73]. Here, EMT tumor cells are induced to become autophagic CSCs in response to cues from the TME. Once these stimuli are relieved and/or are replaced by paracrine factors, autophagic CSCs become cycling (progenitor) CSCs that self-renew and replenish the pool of differentiated tumor cells. Both models foresee the possibility of bidirectional conversion, i.e. from EMT tumor cells to CSCs and vice versa [74]. The latter model is more consistent with a traditional view of stem cell and CSC development and differentiation, while the former is better apt to explain the consequences of regional differences of the TME in giving rise to one or the other CSC subpopulation [60].

## Conclusions

In this article we reviewed the differences between EMT tumor cells and CSCs and proposed two models to explain the cross-talk between EMT tumor cells and two main CSC subpopulations, one non-cycling and autophagic, the other cycling. It is likely, however, that the differences between differentiated tumor cells, EMT tumor cells, and CSC subpopulations are not strict. Thus, it has been demonstrated that EMT tumor cells can exist in different transitions states, from cells with a predominantly epithelial phenotype to cells with a predominantly mesenchymal phenotype [75]. Similarly, it is conceivable that EMT tumor cells acquire characteristics of autophagic CSCs (i.e. activation of autophagy-related gene products) while not entirely losing those of EMT tumor cells, and reacquire predominantly epithelial characteristics when they develop into cycling CSCs. The phenotypic heterogeneity of CSCs supports this possibility [55]. The existence of phenotypic transition states would also imply functional transition states as regards the capacity to invade, migrate and metastasize, resist to apoptosis and antitumor drugs, or to self-renew and differentiate into mature tumor cells.

One key aspect that remains to be addressed concerns the therapeutic consequences deriving from these insights. Given the heterogeneity of the tumor cell population that encompasses differentiated tumor cells, EMT tumor cells, and CSC subpopulations, it appears unlikely that tumor eradication can be achieved without targeting each of these different tumor cell populations. We ignore, however, whether cytotoxic drugs are equally active on differentiated tumor cells and on cycling CSCs. Moreover, we don’t know whether anti-CSC compounds that are in active clinical development [27], including monoclonal antibodies against putative CSC markers [76–78], are equally active on cycling and non-cycling CSCs. Likewise, we don’t know whether compounds that are potentially active on both EMT tumor cells and CSCs are equally active on both populations and on CSC subpopulations. Progress in these directions is warranted. Nevertheless, available knowledge has allowed for the preclinical testing of combination therapies that target some of these tumor cell populations. For instance, while curcumin has antitumor effects but, at the same time, promotes the development of autophagic CSCs, these cells could be depleted by targeting a CSC marker [79]. The combination of a chemotherapeutic drug (temozolomide) targeting cycling tumor cells with a drug targeting quiescent tumor cells has yielded promising results in a genetically engineered mouse model of glioblastoma [80]. Combination of a glycolytic inhibitor targeting glioblastoma CSCs and the cytotoxic drug carmustine significantly impaired the sphere-forming ability of glioblastoma CSCs in vitro and tumor formation in vivo, leading to increase in the overall survival of mice bearing orthotopic inoculation of glioblastoma CSCs [81]. Other similar approaches of combination therapies targeting different tumor cell populations, including CSCs, have been reported [53]. Eventually, as regards autophagic CSCs, it appears logical to test compounds that inhibit autophagy or induce autophagic cell death in combination with drugs that target the cycling tumor cell compartment (mature tumor cells and cycling CSCs). Compounds that inhibit autophagy and that are being tested as anti-CSC compounds in clinical studies are chloroquine or hydroxychloroquine [27, 82, 83], while other compounds of this class are in earlier stages of development [84].

Further preclinical and, eventually, clinical testing of these or forthcoming combination therapies will tell us if our increasing knowledge of EMT and CSC biology can translate into improved therapeutic efficacy.

## Abbreviations

CSC: Cancer stem-like cell
DEDD: Domain-containing DNA-binding protein
EMT: Epithelial-mesenchymal transition
EMT tumor cells: Epithelial-mesenchymal transitioned tumor cell
ESA: Epithelial specific antigen
mTOR: Mammalian target of rapamycin
PDGFR: Platelet-derived growth factor receptor
PI3KC3: Class III phosphatidylinositol 3-kinase
Prrx1: Paired related homeobox 1
TME: Tumor microenvironment
